# Supplementary material for: Lipid-Sensing Receptor FFAR4 Modulates Pulmonary Epithelial Homeostasis following Immunogenic Exposures Independently of the FFAR4 Ligand Docosahexaenoic Acid (DHA)
Source: Int J Mol Sci. 2023 Apr 11;24(8):7072. doi: 10.3390/ijms24087072 (PMC10138935; doi:10.3390/ijms24087072)
Supplement: Supplementary file 1 [file ijms-24-07072-s001.zip › ijms-2283763-supplementary.pdf]

Supplementary Data

S1. a. Plasma fluorescence following DE exposure.

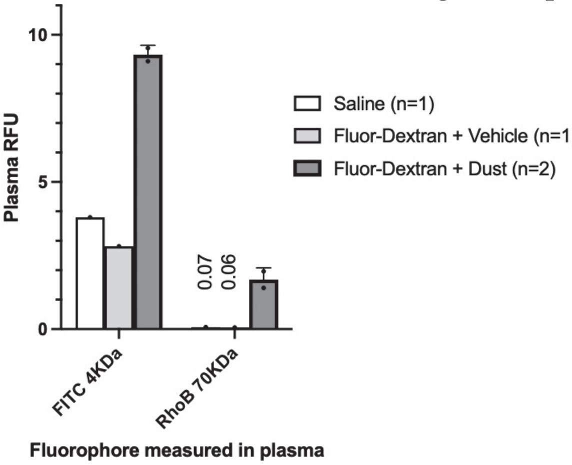

b. Control fluorescence plotted separately.

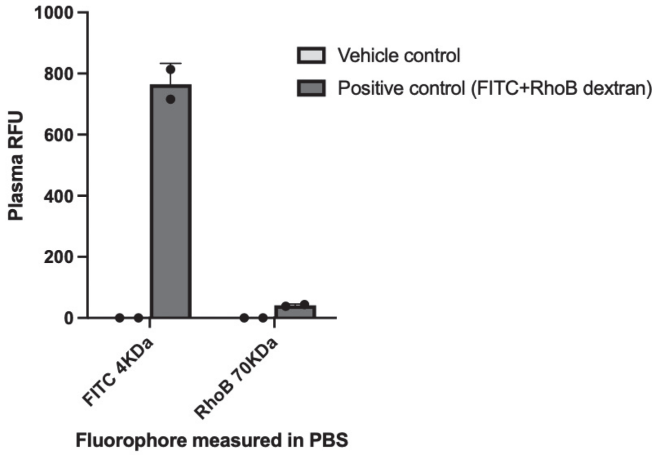

S1. Plasma was collected from wild-type mice that were administered 12.5% dust extract combined with 7mg/kg fluorescent conjugated dextran (FITC and Rhodamine B) for 7 consecutive days and collected on day 7. The plasma fluorescence was quantified using the Thermo Scientific™ Varioskan™ LUX plate reader. A control sample was generated with 5µL each of FITC and RhoB dextran in 100uL PBS.
